# Splicing analysis of *CYP11B1* mutation in a family affected with 11β-hydroxylase deficiency: case report

**DOI:** 10.1186/s12902-016-0118-6

**Published:** 2016-06-17

**Authors:** Pattaranatcha Charnwichai, Patra Yeetong, Kanya Suphapeetiporn, Vichit Supornsilchai, Taninee Sahakitrungruang, Vorasuk Shotelersuk

**Affiliations:** Department of Bioscience, Faculty of Science, Chulalongkorn University, Bangkok, 10330 Thailand; Excellence Center for Medical Genetics, Department of Pediatrics, Faculty of Medicine, Chulalongkorn University, Bangkok, 10330 Thailand; Department of Botany, Faculty of Science, Chulalongkorn University, Bangkok, 10330 Thailand; Center of Excellence for Medical Genetics, King Chulalongkorn Memorial Hospital, Bangkok, 10330 Thailand; Division of Pediatric Endocrinology, Department of Pediatrics, Faculty of Medicine, Chulalongkorn University, Bangkok, 10330 Thailand

**Keywords:** *CYP11B1*, Splicing, Mutation, 11β-hydroxylase deficiency, Congenital adrenal hyperplasia, Case report

## Abstract

**Background:**

Congenital adrenal hyperplasia (CAH) due to steroid 11β-hydroxylase deficiency (11β-OHD) is a rare form of CAH associated with low renin hypertension, hypokalemia, hyperandrogenemia and ambiguous genitalia in affected females. Herein we describe the clinical, hormonal and molecular characteristics of two Uzbekistan siblings with 11β-OHD and analyze the effects of a splicing mutation.

**Case presentation:**

A 46,XX girl presented with genital ambiguity and low renin hypertension; her 46,XY brother presented with precocious puberty. Hormonal studies suggested 11β-OHD. Mutation analysis was performed by PCR followed by Sanger sequencing of the entire coding regions and their flanking introns of the *CYP11B1* gene. Mutation analysis showed that both patients were compound heterozygous for IVS7 + 1G > A, and c.421C > T. Although the identified mutations have been previously described, this is, to our knowledge, the first report of these mutations in compound heterozygotes. A minigene assay was used to determine the effects of the splicing mutation. The constructs containing either the wild-type or the splice-site mutant *CYP11B1* genomic DNA of exons-introns 6–9 were transfected into COS-7 cells; subsequently, RNA splicing was assessed by reversed transcribed-PCR of *CYP11B1* complementary DNA. The minigene assay revealed that the IVS7 + 1G > A mutation resulted in two shorter incorrectly spliced products; one skipping the exon 7 and the other skipping the exons 7–8. The c.421C > T mutation leads to the introduction of a premature stop codon at residue 141 (p.R141X). These mutations are expected to code non-functional proteins.

**Conclusion:**

Compound heterozygous mutations (IVS7 + 1G > A and p.R141X) in the *CYP11B1* gene were found to cause 11β-OHD. The IVS7 + 1G > A mutation causes aberrant splicing of *CYP11B1* leading to exon skipping. This finding could facilitate the future novel therapies targeted on splicing modulation to treat human disease.

## Background

11β-hydroxylase deficiency (11β-OHD) caused by mutations in the *CYP11B1* gene accounts for approximately 5–8 % of congenital adrenal hyperplasia (CAH) in non-consanguineous populations, but accounts for ~15 % of cases in both Muslim and Jewish Middle Eastern populations [1]. Steroid 11β-hydroxylase (P450c11β, *CYP11B1*) converts 11-deoxycortisol to cortisol, representing the final step in cortisol biosynthesis, and 11-deoxycorticosterone (DOC) to corticosterone. Thus, deficient P450c11β activity results in impaired cortisol synthesis and accumulation of 11-deoxycortisol and the mineralocorticoid precursor DOC, which leads to significant hypertension, a hallmark feature of this CAH variant [1]. Accumulated steroid precursors are shunted into the androgen synthesis pathway, leading to androgen excess. Classic 11β-OHD results in virilization of the external genitalia in affected females (46,XX disorders of sex development) as well as precocious puberty, accelerated growth and bone maturation in both sexes. Patients with 11β-OHD can have elevated concentrations of 17-hydroxyprogesterone (17OHP), which accumulates two steps behind the enzymatic block, so that 11β-OHD may be detected by 17OHP newborn screening program [2]. The diagnosis is established by elevated basal concentrations of DOC and 11-deoxycortisol, which hyper respond to ACTH stimulation.

*CYP11B1* is located on the long arm of chromosome 8 (8q21), consisting of 9 exons, and encodes 503 amino acids. To date, over 80 mutations in *CYP11B1* gene are described. Most are missense and nonsense mutations, but splice-site mutations, small or gross deletions/insertions, and complex rearrangements with *CYP11B2* have also been identified [3–5]. The majority of *CYP11B1* mutations are associated with classic 11β-OHD, and only a few mutations causing non-classic 11β-OHD which can manifest later in otherwise asymptomatic women with hirsutism, and menstrual irregularities [6, 7].

In this report, we describe two siblings with the clinical and hormonal phenotypes of 11β-OHD and identified compound heterozygous mutations in the *CYP11B1* gene. The splicing mutation was studied in vitro for its functional consequences with a minigene experiment and showed exon-skipping which confirmed the clinical diagnosis.

## Case presentation

The patients were siblings from a non-consanguineous Uzbekistan family. *Patient 1* was a 3-yr-old 46,XX female with ambiguous genitalia. She was previously evaluated for her abnormal genital development and underwent first genital surgery in Turkey. On an initial evaluation at age 2 y 8 m, her height was 100 cm (+2.1 SD), her weight was 15.8 kg, (+1.4 SD), blood pressure (BP) was 110/70 mmHg (94^th^/96^th^ percentiles). The physical examination revealed that the phallus was 5 cm long and 2 cm wide (Prader grade IV); no gonads were palpable in the inguinal region. The areola and palmar creases were pigmented bilaterally. An ACTH stimulation test (250 μg) showed grossly elevated baseline ACTH (238 pg/mL) and basal cortisol of 4.7 μg/dL with non-response to ACTH and moderately elevated progesterone and 17OHP after 60 min; 11-deoxycortisol and androstenedione concentrations were markedly high (Table 1). The serum sodium was 136 mmol/L, potassium 3.1 mmol/L, plasma renin activity (PRA) was very low at 15 ng/dL/h (nl, 171–1115), and aldosterone 2 ng/dL (nl, 3–35). Her total testosterone levels were 132 ng/dL (nl, <3–10) and dehydroepiandrosterone sulfate (DHEAS) 165.2 μg/dL (nl, <5-57). She was followed up at the King Chulalongkorn Memorial Hospital (Bangkok, Thailand) due to the family relocation at age 3 yr for further management. After receiving the results of an ACTH stimulation test, she was started treatment with hydrocortisone, 5 mg thrice daily (10 mg/m^2^/d) which improved BP into the normal range (90/60 mmHg), suppressed testosterone, and PRA became measurable (200–496 ng/dL/h).

**Table 1:** Basal and 60 min post ACTH (250 μg) stimulated adrenal steroid profile

Steroids	Patient 1		Patient 2		Reference values	
	(age 2 y 8 m)		(age 2 y)			
	Basal	Stimulated	Basal	Stimulated	Basal	Stimulated
ACTH (pg/mL)	238	-	150	-	10–65	-
Cortisol (μg/dL)	4.72	4.65	2.3	2.3	3–22	27–50
17-OHP (ng/dL)	2880	2730	1310	2380	13–173	85–250
Progesterone (ng/dL)	464	499	-	-	^a^	^a^
Androstenedione (ng/dL)	2750	2710	-	-	<10-48	<10–87
11-deoxycortisol (ng/dL)	15100	-	-	-	7-210	95–323

^a^ *Note*: Reference values are unavailable

*Patient 2* is the younger brother of Patient 1. He presented at 2 years of age with acne and masculinization (isosexual precocious puberty). Physical examination revealed an advanced maturation of external genitalia as well as a low-pitched voice. His Tanner stages were G3 and PH1, and each of his testes was 3 mL in volume. His height was 97 cm (+3.2 SD) and weight was 17 kg (+2.9 SD). Height gain was accelerated from 12-month-old on the growth chart (from +2.6 SD to +3.2 SD). Skin pigmentation appeared consistent with his ethnicity, but no evident mucosal pigmentation. His BP was 110/65 mmHg (92^th^/95^th^ percentiles). Labs revealed serum Na 136 mmol/L, K 4.3 mmol/L, bicarbonate 24 mmol/L, BUN 12 and Cr 0.3 mg/dL, respectively. An ACTH stimulation test showed elevated baseline ACTH, a low basal cortisol (2.3 μg/dL) with non-response to ACTH and moderately elevated 17OHP after 60 min (Table 1). 11-deoxycortisol concentrations were not measured. PRA was low at 106 ng/dL/h (nl, 171–1115), and low aldosterone 0.2 ng/dL (nl, 3–35). He was then treated with hydrocortisone 2.5 mg thrice daily (11 mg/m^2^/d). His blood pressure was well controlled, and PRA was increased up to 738 ng/dL/h.

### DNA sequencing

Genomic DNA from peripheral blood leucocytes of the patients and their parents was extracted by using the QIAamp® DNA Blood Mini Kit (Qiagen, Valencia, CA) after taking informed consent. The coding sequence of *CYP11B1* gene including exon-intron boundaries was amplified in eight fragments using specific primers (Table 2). PCR products were treated with *ExoSAP-IT* (USP Corporation, Cleveland, OH), and sent for direct sequencing at Macrogen Inc. (Seoul, Korea). Analyses were performed by Sequencher 4.2 (Gene Codes Corporation, Ann Arbor, MI).

**Table 2:** Sequences of oligonucleotide primers used for PCR amplification and minigene construction

Primer	Sense Strand	Antisense Strand
CYP11B1_Exon 1	5’- GTTCTCCCATGACGTGATCCCTCT – 3’	5’- TCCAAAGGATGCAGAGTGCC – 3’
CYP11B1_Exon 2	5’ – TGGACAGGAGACACTTTGGAT – 3’	5’ – TCGCCGCTTACAGCAAGAAC – 3’
CYP11B1_Exon 3–4	5’ – TGGGGACAAGGAGGATGGGATAC – 3’	5’ – TGGTGGAGAGGGAGAAATTGGG – 3’
CYP11B1_Exon 4	5’ – CGTGGGAAGATCCAGCCTCAG – 3’	5’ – GGAAGGTGAGGAATCCCCGAC – 3’
CYP11B1_Exon 5	5’ – AGGAGGAGGACACTGAAGGATG – 3’	5’ – AGGCAGGCTTGGCATCACC – 3’
CYP11B1_Exon 6	5’ – GGCTCTGTCGTTCTCAGGGTATGC – 3’	5’ – GGCGTTGAAGAGGGATTCCAGAG – 3’
CYP11B1_Exon 7–8 CYP11B1_Exon 9 CYP11B1_IVS7_construct	5’ – AGAGAGCACAGGAAGCCCCATC – 3’ 5’ – GTTCCCCCTTCAGCATAATCTC – 3’ 5’ - AGTCGGATCCCTTGCTGATGACGCTCTTTG - 3’	5’ – CAGTCCCACATTGCTCAAGC – 3’ 5’ – GCCCTCGGGAGTTCCATTT – 3’ 5’- GTACTCTAGAATGGCTCTGAAGGTGAGGAG - 3’

### Minigene construction and splicing analysis

We performed a minigene in vitro experiment of the *CYP11B1* splicing mutation. A segment of the wild-type (WT) and mutant (IVS7 + 1G > A) genomic DNA (gDNA) of *CYP11B1* gene consisting of exons 6 to 9 and their in-between introns was amplified by PCR using the oligonucleotides listed in Table 2. We used the gDNA of a normal control and the patient with IVS7 + 1G > A *CYP11B1* mutation as a template of minigene constructs. PCR reactions were carried out in a 20 μl volume containing 50 ng gDNA, 10xPCR buffer, 25 mM MgCl_2_, 10 μM dNTPs, 5 U/μl *Taq polymerase* and 10 μM of each primer, using the following parameters: 30s at 94 °C, 30s at 60 °C and 1.30 min at 72 °C. The PCR product was cleaved with *BamHI-HF* and *XbaI* enzymes and cloned into the corresponding sites of pcDNA™3.1/myc-His B mammalian expression vector (Invitrogen, Carlsbad, CA) using *T4 DNA ligase* (New England BioLabs, UK). The wild-type and mutant vectors were confirmed by direct sequencing using NCBI Reference Sequences (RefSeq) NG_007954.1 as the genomic reference and NM_000497.3 as the mRNA reference.

COS-7 cells were cultured in Dulbecco’s Modified Eagles Medium, High Glucose (HyClone Laboratories, Logan, UT) supplemented with 10 % fetal bovine serum (Sigma-Aldrich, Singapore) and 0.01 % penicillin/streptomycin (HyClone Laboratories) at 37 °C in a humidified 5 % CO_2_ incubator. Cells were grown on 6-well plates and transiently transfected with the wild-type and mutant minigene constructs (1 μg) using Effectene® Transfection Reagent (Qiagen). Cells incubated for 48 h after transfection and then were washed 3 times with PBS and kept frozen at − 20 °C. Total cellular RNA was extracted using QIAamp® RNA Blood Mini Kit (Qiagen) and treated with *DNaseI* (Qiagen). The RNAs were then used as template for cDNA synthesis using ImProm-II™ Reverse Transcription System (Promega Corporation, Madison, WI). Finally, both the WT and mutant cDNAs were amplified by PCR using the same primers and conditions as used for the minigene construction. The PCR products were analyzed by electrophoresis on a 1 % agarose gel followed by staining with ethidium bromide. Each PCR product was confirmed by Sanger sequencing after subcloning into pGEM®-T Easy vectors (Promega).

## Results

### Mutation analysis

**Fig. 1:**
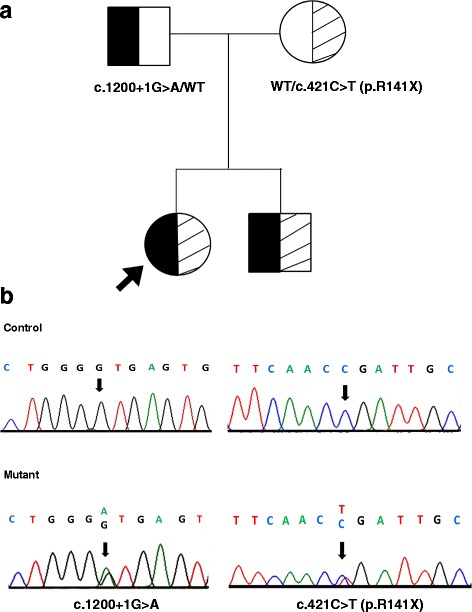
Mutation analysis by direct DNA sequencing. **a** Family tree. The arrow indicates our proband. Both siblings carried compound heterozygous mutations; the point mutation at position bp 421 (c.421C > T) leads to the substitution of arginine to stop at amino acid position 141 (p.R141X), and the base change from G to A at the first position in intron 7 (c.1200 + 1G > A or IVS7 + 1G > A). The father is heterozygous for the c.1200 + 1G > A mutation and the mother is heterozygous for p.R141X. **b** Electropherograms of the patient and a healthy control

DNA sequencing of the entire coding regions and their flanking introns of the *CYP11B1* gene showed that both siblings were compound heterozygous for a nonsense mutation c.421C > T in its exon 3 (NCBI RefSeq NG_007954.1), causing the introduction of a premature stop codon at residue 141 (p.R141X); and a splice site mutation, c.1200 + 1G > A which is at the 5’ donor splice site of the intron 7 (IVS7 + 1G > A). These identified mutations were reported previously [8–10], but their pathogenic mechanisms have not clearly been elucidated. Sequence analysis of the parental gDNA demonstrated that the mother was heterozygous for the c.421C > T mutation and the father, heterozygous for the c.1200 + 1G > A mutation (Fig. 1).

### Minigene analysis of the splice site mutation

**Fig. 2:**
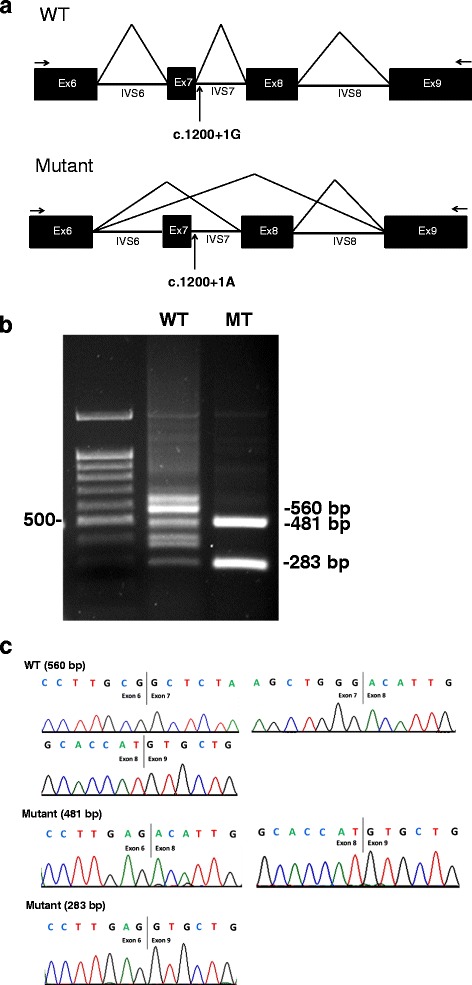
Minigene experiment. **a** The scheme shows the set-up of the minigene constructs for the splicing analysis in the WT and mutant expression vectors containing c.1200 + 1G > A mutation (arrows). **b** COS-7 cells were transfected with the wild-type (WT) or mutant (MT) minigene constructs. Total RNA from the transfected cells were used for RT-PCR of *CYP11B1* cDNA. The figure shows the expected 560-bp PCR product from the WT construct and two shorter incorrectly spliced products from the mutant, sized 481 and 283 bp on an agarose gel. **c** Electropherograms of the minigene PCR products. The 481 bp mutant fragments skipped the entire exon 7, while containing the full-length sequences of exons 6, 8, 9. The 283 bp mutant PCR product skipped the exons 7 and 8, while retaining full-length sequences of exons 6 and 9. Black lines indicated exon–exon boundaries

We hypothesized that the c.1200 + 1G > A (IVS7 + 1G > A) mutation located at the splicing donor site of intron 7 would create an abnormal splicing of the mRNA. To examine this possibility, we constructed expression vectors containing the WT and the mutant c.1200 + 1G > A *CYP11B1* minigene sequence from exons 6 to 9 (Fig. 2a). The resultant minigenes were transfected into COS-7 cells. Then, total RNA was isolated and analyzed by the RT-PCR method. We found that the mutant c.1200 + 1G > A *CYP11B1* minigene was processed to two major incorrectly spliced products which were shorter than the WT minigene (Fig. 2b). Sequence analysis of the RT-PCR products after subcloning into pGEM®-T Easy vector revealed that one of the mutant fragments skipped the entire exon 7, while containing the full-length sequences of exons 6, 8, 9. The other mutant PCR product skipped the exons 7 and 8, while retaining full-length sequences of exons 6 and 9 (Fig. 2c).

## Discussion

In this study, we have described two severe *CYP11B1* mutations found in two siblings diagnosed with classic 11β-OHD in a family from Uzbekistan. Virilization and hypertension are the main clinical features of the classic 11β-OHD. Despite inability of aldosterone synthesis, overproduction of DOC, which is a less potent mineralocorticoid, causes salt retention and hypertension. However, affected newborns may have mild, transient salt loss presumably due to relatively high mineralocorticoids resistance in the newborn period [11]. Signs of mineralocorticoid excess generally correlate poorly with the degree of virilization in affected girls [3]. Blood pressure is usually normal during infancy and hypertension is often identified later in toddlerhood or in childhood, although its presence in infancy was demonstrated [12].

Most of the *CYP11B1* mutations described to date result in the classic form of 11β-OHD. Unlike 21-hydroxylase deficiency, molecular-genetic studies of 11β-OHD are relatively fewer, and a number of identified *CYP11B1* mutations have not been functionally characterized [5, 13]. Therefore, the exact genotype-phenotype prediction of 11β-OHD has not been well established. Previous studies showed that in vitro activities less than 5 % were considered severe and consistent with classic 11β-OHD [5, 13]. In this present study, we describe two siblings suffering from classic 11β-OHD who were compound heterozygous for a nonsense and a splice-site *CYP11B1* mutations. The nonsense p. R141X is expected to lead to a premature stop in the exon 3 and yields a truncated enzyme lacking the essential residues for heme binding domain, consistent with our patients’ clinical phenotypes and near-completely abolished in vitro CYP11B1 activity in a recent study [14].

In addition, we identified a previously described IVS7 + 1G > A mutation in *CYP11B1* affecting the consensus slice donor site of the exon 7. The minigene experiment confirmed that this splice site mutation caused exon skipping (either a complete loss of the exon 7 or both exons 7 and 8). Most reported *CYP11B1* mutations are located in exons 6, 7, and 8 and 70 % of amino acid sequences in these exons are identical in human, ox, rat, and mouse, suggesting that exons 6–8 are essential for the enzymatic activity of CYP11B1 [15]. Recently, Nguyen et al. [9] studied a minigene experiment of this same mutation. Nonetheless, the authors designed a shorter minigene construct which had only exon 7, intron 7, and exon 8. They found that the IVS7 + 1G > A mutation caused an intron retention.

Splicing errors are well recognized causes of genetic diseases. Previous data point to an estimated frequency of sequence variations affect pre-mRNA splicing up to 50 % of the alleles causing human disease [16, 17]. Splice site nucleotide substitutions may result in skipping of the involved exon, intron retention, creation of a pseudo-exon within intron, usage of a cryptic splice site, or a combination of several of these [18, 19]. Hence, the design of minigene constructs is important to correctly identify the splicing effect of specific splice site mutations. Recent data have suggested that cassette exon skipping is the most common alternative splicing event in humans [19, 20]. To date, a +1G > A substitution at the 5’-splice donor site has been identified in a number of human diseases [21]. Functional studies of other +1G > A 5’-splice site mutations have shown either recognition of a 5’-cryptic splice site or exon skipping [22]. Therefore, we have designed the minigene constructs including exons 6–9 and introns 6–8 and our results confirmed that the IVS7 + 1G > A mutant construct results in exon skipping. To date, the therapies to modulate RNA mis-splicing using antisense oligonucleotide or small molecules are emerging [19]. The understanding of definite genetic mechanism could expand opportunities for gene therapy. Modulation of aberrant splicing transcripts can become a novel therapeutic approach for many diseases caused by splice site defects.

## Conclusions

In summary, we describe two compound heterozygous *CYP11B1* mutations that severely affect normal protein structure explaining a severe phenotype of classic 11β-hydroxylase deficiency. Our findings suggest the mutation IVS7 + 1G > A causes aberrant splicing of *CYP11B1* leading to exon skipping. Our findings may help for better understanding of splice site mutation mechanism and facilitate the future new therapies targeted on splicing modulation to treat human disease.

## Abbreviations

11β-OHD, 11β-hydroxylase deficiency; 17OHP, 17-hydroxyprogesterone; ACTH, adrenocorticotropic hormone; CAH, congenital adrenal hyperplasia; DHEAS, dehydroepiandrosterone sulfate; DOC, 11-deoxycorticosterone; gDNA, genomic DNA; mRNA, messenger RNA; MT, mutant; nl, normal; PCR, polymerase chain reaction; PRA, plasma renin activity; RT-PCR, reverse transcription polymerase chain reaction; WT, wild-type
